# Incipient Diabetes Mellitus and Nascent Thyroid Disease Presenting as Beard Alopecia Areata: Case Report and Treatment Review of Alopecia Areata of the Beard

**DOI:** 10.7759/cureus.9500

**Published:** 2020-07-31

**Authors:** Parnia Forouzan, Philip R Cohen

**Affiliations:** 1 Dermatology, McGovern Medical School, University of Texas Health Science Center at Houston, Houston, USA; 2 Dermatology, San Diego Family Dermatology, National City, USA

**Keywords:** alopecia, areata, beard, diabetes, hair, incipient, loss, mellitus, thyroid, treatment

## Abstract

Alopecia areata is a non-scarring hair loss that commonly presents on the scalp. In men, when this condition results in facial hair loss on the cheek, jaw, and neck, it is referred to as beard alopecia areata. Beard alopecia areata can be associated with autoimmune conditions, such as diabetes mellitus, thyroid disorders, and vitiligo. A 28-year-old man presented with a five-month history of facial hair loss; his condition was diagnosed as beard alopecia areata after clinical examination. Treatment with twice daily topical 0.1% triamcinolone acetonide cream led to complete regrowth of his beard hair after six months. There are several potential agents and modalities for the treatment of individuals with beard alopecia areata. Treatment options include corticosteroid therapy (intralesional or topical), immunotherapy, Janus kinase (JAK) inhibitors, lasers, photodynamic therapy, platelet-rich plasma therapy, and treatment of an underlying *Helicobacter pylori* infection. Laboratory evaluation, prompted by our patient’s diagnosis of beard alopecia areata, suggested incipient diabetes mellitus and nascent thyroid disease; specifically, he had elevated fasting blood glucose and elevated thyroid-stimulating hormone levels. Therefore, in patients with beard alopecia areata, laboratory evaluation for concomitant or incipient autoimmune diseases should be considered.

## Introduction

Alopecia areata is a non-scarring type of alopecia. It usually appears as small, annular patches of hair loss on the scalp; however, more severe variants of alopecia areata can occur: complete scalp hair loss (alopecia totalis) or total body hair loss (alopecia universalis). Occasionally, alopecia areata affects facial hair; in men, this can present as hair loss in the beard area [1].

Alopecia areata is an autoimmune disease. It can occur as an isolated condition or be associated with other autoimmune disorders. The comorbid conditions potentially associated with alopecia areata include diabetes mellitus, psoriasis, thyroid disorders, and vitiligo [2].

A 28-year-old man presented with beard alopecia areata. His alopecia areata completely resolved with topical corticosteroid treatment. Subsequent laboratory evaluation revealed not only incipient diabetes mellitus but also nascent thyroid disease. The features of beard alopecia areata, emphasizing comorbidities and management, are summarized.

## Case presentation

A 28-year-old man presented with a five-month history of hair loss on the beard area. He had no other symptoms. However, his family history was significant for diabetes mellitus.

**Figure 1:**
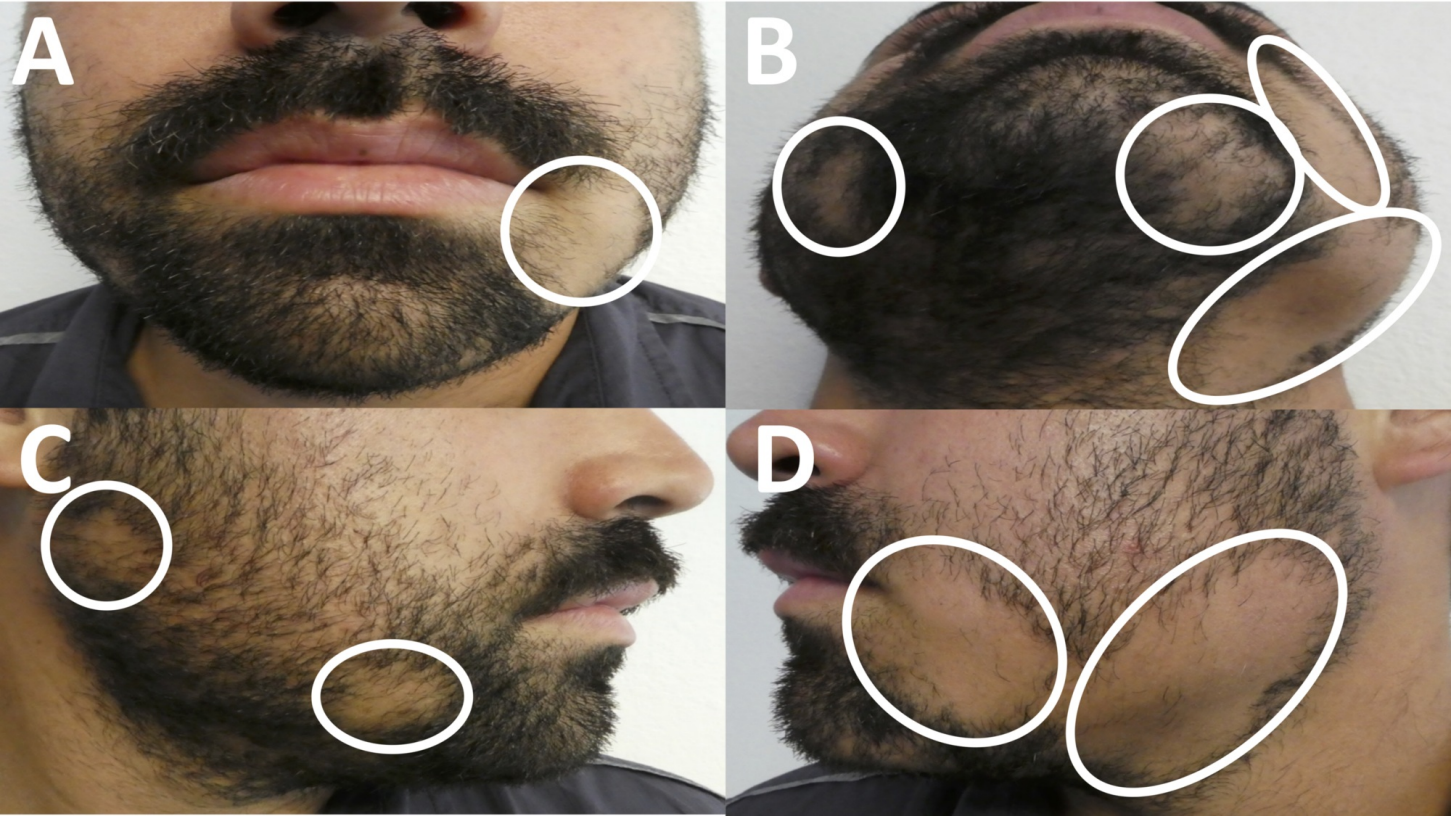
Clinical presentation of beard alopecia areata Frontal (A and B), right-sided (C), and left-sided (D) views of a 28-year-old man’s beard with annular patches of hair loss (circled in white).

Cutaneous examination revealed two larger patches of hair loss on the left jaw and cheek; there was also a smaller patch of hair loss on his left chin. In addition, there were two smaller patches of hair loss on the right jaw and cheek (Figure 1).

A diagnosis of beard alopecia areata was established based on the clinical history and the morphologic appearance of his hair loss. Intralesional triamcinolone acetonide injections and topical corticosteroid use were discussed as possible treatment options. He decided to pursue therapy with twice daily application of topical 0.1% triamcinolone acetonide cream on the areas of hair loss.

Laboratory evaluation was performed to evaluate him for comorbid autoimmune conditions that can potentially be associated with alopecia areata. Normal or negative results were found for antinuclear antibodies (ANA), rheumatoid factor (RF), and thyroxine (T4). However, his fasting blood sugar (glucose) level was elevated at 103 mg/dL (normal, 65-99 mg/dL), and his thyroid-stimulating hormone (TSH) level was elevated at 6.4 mIU/L (normal, 0.4-4.5 mIU/L). Repeat laboratory studies by his primary care physician confirmed the initial observations of elevated fasting blood sugar and TSH levels.

He was treated with dietary management for his elevated, pre-diabetic blood sugar levels. He had no clinical symptoms of hypothyroidism; therefore, he did not receive any medical intervention for his elevated TSH level. Periodically, he will continue to have follow-up with his primary care physician to monitor his blood sugar and thyroid function.

**Figure 2:**
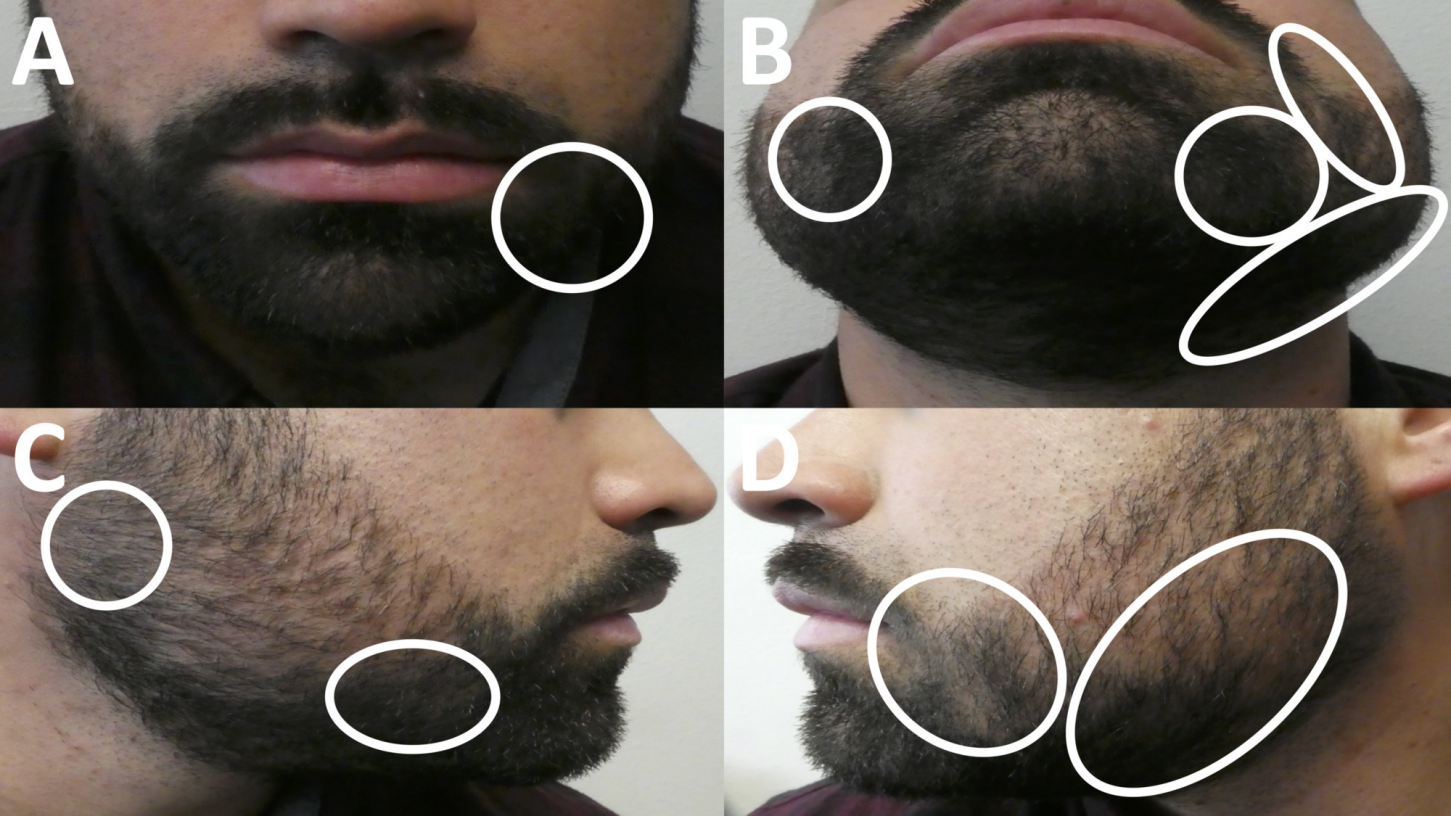
Clinical presentation of alopecia areata of the beard following six months of topical corticosteroid treatment Views of complete regrowth of beard hair in areas of previous alopecia areata (circled in white) from the front (A and B), right side (C), and left side (D) after treatment with twice daily topical 0.1% triamcinolone acetonide cream for six months.

The patient presented for dermatology follow-up of his beard alopecia areata at one, two, and six months after his initial visit. Early beard hair regrowth was seen at the two-month follow-up visit. Complete hair regrowth was observed at his six-month follow-up visit (Figure 2). Therefore, after six months of twice daily treatment, he decreased the use of the triamcinolone acetonide cream each month to once daily, followed by three times weekly, and finally twice weekly before stopping its use.

## Discussion

Beard alopecia areata can occur alone or in concert with other areas of alopecia areata-associated hair loss. Following alopecia areata of the scalp, alopecia areata of the beard is the most common site of hair loss in men; it occurs in 28% of men with alopecia areata. The diagnosis of beard alopecia areata is typically based on the clinical examination of the patient. Clinically, it presents with well-circumscribed, annular patches of hair loss on the cheeks, jaw, and neck [1].

Dermoscopy findings of beard alopecia areata commonly include black dots, hairs that taper at the base (described as “exclamation point hairs”), regrowing hair, short vellus hair, white dots (due to the absence of hair follicles), and yellow dots (due to the collection of sebum and keratin). Microscopic examination of alopecia areata on the scalp or the beard reveals lymphocytes around the hair follicle shaft (described as a “swarm of bees”), leading to weakening of the hair shaft and hair loss. There is no scarring or fibrosis associated with alopecia areata; therefore, the potential for future hair regrowth exists [1,2].

Associated diseases may be present in men with beard alopecia areata. For example, our patient had incipient diabetes mellitus and nascent thyroid disease. Other associated conditions with alopecia areata include atopic dermatitis, celiac disease, pernicious anemia, psoriasis, rheumatoid arthritis, systemic lupus erythematosus, and vitiligo [3,4].

Individuals with alopecia areata have a 16% greater risk for concurrently having or subsequently developing other autoimmune conditions [1]. Therefore, laboratory examination should be considered in patients with beard alopecia areata. Blood tests to perform may include evaluation for autoantibodies (ANA, double-stranded DNA [dsDNA] antibody, ribonucleoprotein [RNP] antibody, SCL70 antibody, Smith [Sm] antibody, Sjögren syndrome A [SSA]/Ro antibody, Sjögren syndrome B [SSB]/La antibody, and thyroid antibodies, such as microsomal, peroxidase, and thyroglobulin antibodies), fasting blood sugar (glucose), hemoglobin A1c (percent), RF level, and thyroid function (TSH, triiodothyronine [T3], and thyroxine [T4]). Our patient’s laboratory evaluation discovered not only incipient diabetes mellitus but also nascent thyroid disease.

Multiple therapies have been identified as effective treatments for beard alopecia areata (Table 1) [3-14]. Treatment with corticosteroids (either intralesional or topical) is usually the first line of therapy. Janus kinase (JAK) inhibitors, lasers, minoxidil, photodynamic therapy, and topical immunotherapy may be used for men who have more resistant or widespread hair loss [5,6].

**Table 1:** Treatment options for beard alopecia areata CR: current report; JAK: Janus kinase

Treatment	References
Immunosuppressants	[5,7], CR
Corticosteroids (intralesional injections, topical)	[5,7], CR
Immunotherapy	[6]
Anthralin and diphenylcyclopropenone	[6]
Janus kinase (JAK) inhibitors	[3,8,9]
Ruxolitinib	[8]
Tofacitinib	[3,9]
Laser	[10-12]
308-nm excimer laser	[10]
908-nm diode laser	[11]
1,550-nm fractional laser	[12]
Minoxidil	[7]
Photodynamic therapy	[13]
Platelet-rich plasma (PRP)	[14]
Prostaglandin F2-alpha analogues	[7]
Latanoprost	[7]
Treatment of concurrent Helicobacter pylori infection	[4]

Our patient decided to treat his beard alopecia areata with topical 0.1% triamcinolone acetonide cream. This led to a complete regrowth of his beard hair within six months. Side effects with topical corticosteroid use are usually minimal and may include folliculitis [7].

Intralesional triamcinolone acetonide injections are an alternative early management for beard alopecia areata. In our clinical experience, dosages between 3 and 5 mg/mL of triamcinolone acetonide may be adequate. One study of 83 patients with beard and/or scalp hair loss found that the optimal treatment for beard alopecia areata was three to four injections, one per month, using a low dose of 0.1 mg/mL triamcinolone acetonide. Approximately 85.7% of individuals experienced at least 75% beard and scalp hair regrowth within six months. Higher doses of intralesional triamcinolone acetonide injections can be associated with folliculitis, pustule formation, skin atrophy, and telangiectasias [5].

Topical immunotherapy, using an irritant agent such as anthralin, diphenylcyclopropenone, or squaric acid dibutyl ester, can be used to elicit an allergic contact dermatitis when applied to the skin. A proposed mechanism of action for this intervention involves diverting the immune response away from hair follicles and thereby allowing for hair regrowth. Topical immunotherapy has become a common treatment for extensive alopecia areata or recurring alopecia areata for patients in whom corticosteroids are ineffective [6].

In a study of 52 men and women with severe alopecia or corticosteroid therapy-resistant alopecia, 12 men had beard hair involvement. Treatment with a combination of diphenylcyclopropenone and anthalin led to complete regrowth of beard hair in 85.7% of men. However, diphenylcyclopropenone treatment alone did not allow for complete beard hair regrowth in this study [6].

The diphenylcyclopropenone and anthralin combination treatment regimen began with the application of 0.001% diphenylcyclopropenone to areas of hair loss. The agent remained on the skin for 48 hours before being washed off. The following five days, 0.5% anthralin was applied for 10 minutes daily before washing off. The concentrations of diphenylcyclopropenone and anthralin were increased weekly until an irritant dermatitis was observed with treatment. Bullae or pruritus was seen in all patients. In addition, folliculitis, hyperpigmentation, and lymphadenopathy were noted in some individuals [6].

JAK inhibitors are an option for treatment-resistant alopecia areata of the beard. JAK is an enzyme involved in inflammatory and immune proliferative pathways. JAK inhibitors have been used to treat skin conditions such as psoriasis and vitiligo. Ruxolitinib and tofacitinib are JAK inhibitors that have been efficacious at specifically treating alopecia areata of the beard [3,8,9].

A 33-year-old man presented with an 11-year history of hair loss resulting in alopecia universalis that was minimally responsive to short-term intralesional and systemic corticosteroid therapy. Treatment with 20 mg ruxolitinib twice daily led to complete regrowth of his beard hair after four months. At the one-year follow-up, full regrowth of his beard hair and 50% regrowth of his scalp hair were still observed. He had no side effects from the treatment [8].

In a retrospective review of 45 patients with beard alopecia areata, encouraging results were observed with daily oral tofacitinib therapy with a mean dose of 7.2 mg. In an average of 16 months, 10 men exhibited complete beard hair regrowth and 19 men achieved partial beard hair regrowth. Adverse events included elevated liver enzymes, fatigue, and mild upper respiratory tract infections in 10 of the men [9].

Two men presented with corticosteroid treatment-resistant alopecia universalis, which included hair loss on the beard area. Both men were successfully treated with twice daily 5 mg tofacitinib. Significant beard hair regrowth was observed in four months with no serious side effects from the treatment [3].

Minoxidil, a vasodilatory agent, has been used to treat alopecia areata of the beard. Minoxidil is thought to increase delivery of blood and nutrients to hair follicles, promoting their growth and strength. In addition, it can stimulate the proliferation of hair follicles [7].

In one study of 100 patients with alopecia areata, one group of 16 men and 4 women were treated with topical 5% minoxidil lotion applied twice daily for up to 20 weeks. The investigators noted a 42.6% improvement in hair regrowth in men with beard alopecia areata. In addition, full regrowth of hair was observed in eight individuals with scalp and/or beard alopecia areata. However, 6 of the 20 patients in this treatment group reported itching with topical minoxidil therapy [7].

Phototherapy has successfully been used to treat skin conditions, such as psoriasis and vitiligo. Light therapy has also been investigated for the treatment of hair loss. The use of lasers or photosensitive agents and light can lead to hair regrowth in beard alopecia areata [10-13].

A study using a 308-nm excimer laser was conducted. Seven men had 10 lesions of beard alopecia areata. Four of the ten lesions experienced hair regrowth with the laser therapy; this required up to 24 treatments [10].

During the treatments, only minimal cutaneous side effects were noted, including mild erythema, hyperpigmentation, and pruritus of the skin. An observation during this study was that the six lesions with no response were from three patients with concurrent asthma. The investigator speculated that the unresponsive lesions may be more treatment resistant due to elevated levels of inflammatory cells, cytokines, and mediators associated with asthma [10].

A 904-nm diode laser has also shown efficacy in treating alopecia areata of the beard. In one study, 11 of 12 lesions from five patients had complete hair regrowth after weekly laser sessions for one month. There were no significant side effects reported [11].

A patient with beard alopecia areata was treated monthly using a non-ablative 1,550-nm fractional laser. After one treatment, more than 50% regrowth of hair was observed. After three months, there was more than 75% hair regrowth; all of his beard hair remained after three years of follow-up. His only side effect was temporary pain at the laser sites [12].

Photodynamic therapy is a light-based therapy that has been used to treat actinic keratoses. A topical photosensitive agent is applied; when the agent is activated by exposure to specific wavelengths of light, cytotoxic compounds are released and promote cell death where the agent was applied. It has also been effective in treating alopecia areata of the beard in one patient [13].

A 26-year-old man presented with a 14-month history of beard hair loss. His beard alopecia areata was resistant to treatment with intralesional and topical corticosteroids, minoxidil, and topical tacrolimus. He was treated with photodynamic therapy, which included topical application of methylaminolevulinic acid, a photosensitizer, for three hours followed by 630-nm red light irradiation for 7.5 minutes [13].

After four sessions of photodynamic therapy, he experienced complete regrowth of his beard hair. The only side effect observed was temporary erythema. However, none of the other five men in the study undergoing the same treatment for scalp alopecia areata experienced complete hair regrowth [13].

Platelet-rich plasma therapy can be anti-inflammatory. It is currently utilized in the management of androgenic alopecia. The plasma is obtained from the top layer of the patient’s centrifuged blood sample and placed into areas of hair loss [14].

A 30-year-old man with a two-year history of beard alopecia areata was successfully treated with three injections of platelet-rich plasma at six-week intervals. Complete hair regrowth was observed at the one-year follow-up. Adverse effects included pain at the injection sites, but the discomfort faded within two days [14].

Prostaglandin F2-alpha analogues have been investigated for the treatment of hair loss. They contract smooth muscle in blood vessels, bronchi, and the uterus. Analogues such as bimatoprost and latanoprost have been used to treat glaucoma. Side effects seen with their use include hyperpigmentation and hypertrichosis of the eyelashes [7].

One study involving 12 men and 8 women with scalp and/or beard alopecia areata found twice daily topical application of 0.1% latanoprost on areas of hair loss for up to 20 weeks to be an efficacious treatment. There was a 42% improvement of hair regrowth in lesions of beard alopecia areata after treatment. In the study of 20 men and women with scalp and/or beard alopecia areata, 20% observed complete hair regrowth with latanoprost therapy. Burning and itching were noted in 10% of the patients [7].

In one patient, treatment of a concurrent condition lead to hair regrowth. A 43-year-old man presented with an eight-month history of alopecia areata of the beard and scalp. He was treated topically with 0.25% desoximetasone and 5% minodixil without improvement. Investigation of his concurrent dyspepsia led to the discovery of an underlying infection with *Helicobacter pylori*. *H. pylori* infection has been associated with not only gastric disorders but also psoriasis, Sjögren’s syndrome, and other autoimmune conditions [4].

Treatment with twice daily 1,000 mg amoxicillin, 500 mg clarithromycin, and 20 mg omeprazole for his *H. pylori *infection led to its eradication. Hair regrowth began four weeks after the treatment period. Complete hair regrowth was observed 16 weeks after finishing his treatment regimen [4].

In our review of the literature, we found other treatments that were successful for alopecia areata of scalp; however, the investigators did not mention the use of these agents for beard alopecia areata (Table 2) [1,15-19]. It is reasonable that these treatment modalities can be considered for patients with refractory or recurrent beard alopecia areata. However, the efficacy of these treatments for alopecia areata of the beard has not been established.

**Table 2:** Treatment options for scalp alopecia areata AA: alopecia areata; AT: alopecia totalis; AU: alopecia universalis; AZA: azathioprine; Cs: corticosteroid; MOP: methoxypsoralen; MTX: methotrexate; OCs: oral corticosteroid; PUVA: psoralen and ultraviolet A; RA: retinoic acid; Ref: reference; Top: topical; UV: ultraviolet; UVA: ultraviolet A.

Agent	Background	Comments	Ref
AZA	AZA is an antimetabolite drug that inhibits purine synthesis and can be used as an immunosuppressive agent.	In a study of four patients, 1 mg/kg AZA was effective in treating AA of the scalp. Noticeable hair regrowth of the scalp was seen in all of the patients. Side effects included myelosuppression, nausea, and pancreatitis.	[15]
MTX	MTX is an immunosuppressive agent that acts by inhibiting nucleotide synthesis.	In an analysis of more than 400 individuals with AA taking MTX, complete hair regrowth was observed in 36% of patients. More than 50% hair regrowth was observed in 63% of patients. In addition, a combination of MTX and Cs therapy was two times more likely to result in greater than 50% scalp hair regrowth. Notable side effects included hematologic insufficiency and liver dysfunction.	[16]
OCs	In AA refractory to topical or intralesional Cs treatments, OCs may be used. Pulsed Cs therapy is effective in 60% of patients with AA.	In one study, monthly oral 300 mg prednisolone allowed for complete or substantial hair growth in 14 of 24 patients with AA or AT. Hair growth was observed after two months with substantial improvement in four months. Side effects were minimal with this dosage, but can include mood changes, polyuria, and weight gain at higher doses.	[1,17]
RA	RA can play a role in hair follicle formation, inhibition, and patterning. Adapalene, a topical retinoid analogue, is commonly used to treat acne.	When 0.1% adapalene gel and 0.1% mometasone furoate (Cs) cream were used in combination once daily for 12 weeks, an average of 90.5% hair regrowth of the scalp was observed. No side effects were reported. Similar treatment with twice daily application of 0.1% mometasone furoate cream alone allowed for 71% hair regrowth of the scalp.	[18]
Top PUVA	PUVA treatment has been used to treat AA of the scalp. Management includes pretreatment with topical psoralen in areas of hair loss followed by exposure to UVA light. The UV light destroys the inflammatory lymphocytes that are causing hair loss.	In one study, 56% of patients with AT or AU observed hair regrowth with PUVA treatment. In addition, 84% of patients with AA experienced hair regrowth. The treatment regimen included application of 0.1% 8-MOP solution to the scalp 20 minutes before a 12 J/cm^2^ dose of UVA light was applied. Patients were treated every three months until hair regrowth was observed. Side effects such as erythema of the skin were reported, and four patients experienced a burning sensation at the treatment sites.	[19]

Notably, not all immunosuppressants have been effective in treating alopecia areata. Calcineurin inhibitors are immunosuppressive agents that inhibit interleukin-2 dependent activation of T cells in the immune response. Calcineurin inhibitors, such as cyclosporine or tacrolimus, have been effective in treating vitiligo, an associated condition of alopecia areata; however, their effectiveness has not been demonstrated for treating alopecia areata of the scalp and/or beard. Indeed, a 26-year-old man with beard alopecia areata had no clinical benefit with tacrolimus treatment; however, he did observe hair regrowth with photodynamic therapy consisting of topical application of methylaminolevulinic acid followed by 630-nm red light irradiation [13,20].

## Conclusions

Alopecia areata of the beard is a lymphocyte-mediated autoimmune condition that results in annular, non-scarring hair loss. Other autoimmune conditions, such as diabetes mellitus, psoriasis, thyroid disorders, and vitiligo, may occur concurrently or subsequently in patients with beard alopecia areata. The front line of treatment for beard alopecia areata is topical corticosteroids; other routes of administration (such as injection or oral) may be used as well. Topical immunotherapy is also commonly used to treat alopecia areata of the beard. Other therapies that have been used in beard alopecia areata include JAK inhibitors, lasers, minoxidil, photodynamic therapy, platelet-rich plasma therapy, and prostaglandin analogues. It is also possible that other modalities used for scalp alopecia areata (such as retinoic acid, systemic immunosuppressants, and topical psoralen with ultraviolet light) may be effective for alopecia areata of the beard but need further investigation.
